# Does seed mass drive interspecies variation in the effect of management practices on weed demography?

**DOI:** 10.1002/ece3.8038

**Published:** 2021-09-02

**Authors:** Elena Kazakou, Guillaume Fried, Pierre‐Olivier Cheptou, Olivier Gimenez

**Affiliations:** ^1^ CEFE Univ Montpellier CNRS EPHE, IRD Univ Paul Valéry Montpellier 3 Institut Agro Montpellier Supagro Montpellier France; ^2^ Anses Laboratoire de la Santé des Végétaux Unité Entomologie et Plantes Invasives Montferrier‐sur‐Lez France; ^3^ CEFE Univ Montpellier CNRS EPHE, IRD Univ Paul Valéry Montpellier 3 Montpellier France

**Keywords:** Bayesian hidden Markov models, plant demography, seed bank, seed size, soil management practices

## Abstract

Optimizing the effect of management practices on weed population dynamics is challenging due to the difficulties in inferring demographic parameters in seed banks and their response to disturbance. Here, we used a long‐term plant survey between 2006 and 2012 in 46 French vineyards and quantified the effects of management practices (tillage, mowing, and herbicide) on colonization, germination, and seed survival of 30 weed species in relation to their seed mass. To do so, we used a recent statistical approach to reliably estimate demographic parameters for plant populations with a seed bank using time series of presence–absence data, which we extended to account for interspecies variation in the effects of management practices on demographic parameters. Our main finding was that when the level of disturbance increased (i.e., in plots with a higher number of herbicides, tillage, or mowing treatments), colonization success and survival in large‐seeded species increased faster than in small‐seeded species. High disturbance through tillage increased survival in the seed bank of species with high seed mass. The application of herbicides increased germination, survival, and colonization probabilities of species with high seed mass. Mowing, representing habitats more competitive for light, increased the survival of species with high seed mass. Overall, the strong relationships between the effects of management practices and seed mass provide an indicator for predicting the dynamics of weed communities under disturbance.

## Graphical abstract

This is one of the very few studies determining the effect of management practices on seed bank dynamics in weed species, and notably the first one ever in vineyards. We used a long‐term plant survey between 2006 and 2012 in 46 vineyard plots in France (Champagne, Beaujolais, and Languedoc wine‐growing areas) with 883 flora surveys to test whether interspecific variation in the effects of management practices (tillage, mowing, and herbicide use) on demographic parameters could be explained by seed mass. We used a hidden Markov model (HMM) recently developed to estimate colonization, germination, and seed bank survival using presence–absence observations which we extended with a multilevel model in a Bayesian framework. The strong relationships between the intensity of the management effects and seed mass provide a reliable indicator for predicting the dynamics of weed communities.

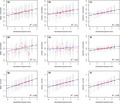

## 1 INTRODUCTION

Managing weeds is one of the most challenging issues faced by farmers, as weeds can cause significant reductions in crop growth and yields, especially in resource‐limited agroecosystems (Hembree & Lanini, 2006). In vineyards, conventional weed control methods rely on different combinations of herbicide applications, soil tillage, and/or mowing applied in the vine rows and the area between them (inter‐rows) (Steinmaus et al., 2008). In the last decades, due to the intensive management of vineyards, several ecosystem services have been affected, causing high rates of soil erosion, degradation of soil structure and fertility, contamination of groundwater, and high levels of agricultural inputs (Zaller et al., 2014). The potential negative side effect of herbicides on food safety, public health and the environment (e.g., Richards et al., 1987) on one side, the herbicide resistance (e.g., Moss, 2003) and stricter regulations with regard to some molecules (e.g., Sass & Colangelo, 2006) on the other side, have led to a consideration of ecological weed management practices.

To optimize weed management, we need to not only quantify weed species presence and abundance, but also to understand species demography. Seed bank is known to have a major impact on plant dynamics (Bekker et al., 1998). An intimate understanding of traits affecting seed bank dynamics such as seed dormancy, seed longevity, and seedling emergence is necessary to determine weed community responses to different soil management practices (Forcella et al., 1993). However, due to the high level of spatial heterogeneity, the time and effort required to conduct adequate sampling (germination chambers, seed separation) the direct estimation of seed banks remains challenging (Louvet et al., 2021). Therefore, models using occupancy data above‐ground can provide information about the presence of the species below‐ground through the existence of a seed bank (Borgy et al., 2015; Fréville et al., 2013). A hidden Markov model (HMM) was recently developed to estimate colonization, germination, and seed bank survival from 1 year to the next without addition of new seeds, using above‐ground presence–absence observations (Pluntz et al., 2018). Here, we extend this HMM with a multilevel model in a Bayesian framework (e.g., Qian et al., 2010) (aka hierarchical Bayesian modeling) to determine interspecies variation in the effects of management practices on weed demography at both the plot and species level, while explicitly considering the seed bank.

Disturbance and more precisely seed burial affects demographic parameters and by consequence vegetation structure and composition (Fried et al., 2019). Generally, emergence inhibition increases proportionately with depth of seed burial in soil for most weed species (Benvenuti et al., 2001; Gardarin et al., 2010). Weed seed survival in soil is also a function of burial depth. Seeds near the soil surface generally die more quickly than those buried more deeply (Mohler & Galford, 1997), because seeds on the surface may be subject to greater losses due to greater activity densities of seed predators (e.g., Kulkarni et al., 2015). Additionally, seeds at the surface have a favorable contact with oxygen, which is one of the main factors involved in aging and loss of vitality in seeds (Hendry, 1993).

Among management practices, mechanical weeding and especially tillage, affect the most weed emergence and germination, as it is the primary cause of vertical seed movement in agricultural soils (Cousens & Moss, 1990). Germination probability in most cases will increase after mechanical weeding or reduced tillage (McConnaughay & Bazzaz, 1987; McIntyre et al., 1995; Reader, 1993). Additionally, external colonization can be more pronounced in frequently disturbed environments (reduced tillage, or herbicide application) (Turnbull et al., 1999). Repeated chemical control select herbicide‐resistant plants, which have been found to be smaller in size, to have lower germination and to exhibit reduced growth rate (Bravo et al., 2017; Van Etten et al., 2016). While most studies have focused on soil‐mediated disturbance, mowing can also impact weed traits. Mowed plants produce significantly a smaller number of fruits, and a smaller number of total seeds per plant, but have higher seed mass, and germinate more and faster (Chavana et al., 2021).

From a mechanistic perspective, we expect that seed mass, because of its influence on key processes such as dispersal in space *via* colonization and dispersal in time *via* seed bank persistence (Coomes & Grubb, 2003), will affect the influence of management practices on plant demographic parameters. Stored resources in large seeds tend to help the young seedling to survive and establish in the face of environmental hazards (e.g., deep shade, drought). Seed mass may also be related to survival in the soil (Bekker et al., 1998; Thompson, 1987) and to disturbances (McIntyre et al., 1995). In arable land, seed mass negatively correlates with the effects of disturbances: Small‐seeded species have a better chance to escape the effects of frequent disturbance than large‐seeded species that are selected under dense plant cover (Albrecht & Auerswald, 2009). However, increased seed persistence is not always associated with reduced seed size. This is because persistence depends not only on seed size, but on other traits, many of them physiological. In many habitats, the probability of seed burial is strongly linked to seed size and shape (a negative relationship between seed mass and depth of emergence), but in arable habitats cultural practices have broken this link (Benvenuti et al., 2001).

Here, we present a model, which aims to quantify the effects of management practices on three major demographic parameters: germination probability, the joint probability of seed germination success and of plant survival to adulthood; seed survival probability in the seed bank, the probability of seed bank survival from 1 year to the next without the addition of new seeds; and external colonization probability, the probability that at least one seed from outside arrived on the plot and survived to the onset of the next season. Our first hypothesis is that frequent disturbances will increase external probability that at least one seed from outside arrived on the plot and survived to the onset of the next season (hereafter external colonization probability) and this effect will be more important for large‐seeded species (Thomson et al., 2010). Our second hypothesis is that frequent disturbance will affect demographic parameters and that this effect will be more intense according to species seed mass. Our third hypothesis is that the frequency of disturbance will increase seed survival in the soil as seedling of large‐seeded species should better survive hazards like deep shade or physical damage (Westoby et al., 1992).

To explore these hypotheses, we used a unique data set covering 46 vineyard plots in France (Champagne, Beaujolais, and Languedoc wine‐growing areas) with 883 flora surveys performed between 2006 and 2012. First, we used our novel multilevel HMM model to test the effects of environmental variables on germination, colonization and seed persistence in the seed bank. Second, we tested whether interspecific variation in the effects of management practices (tillage, mowing and herbicide use) on demographic parameters could be explained by seed mass.

## 2 MATERIAL AND METHODS

### 2.1 The Biovigilance dataset

Vegetation surveys were conducted in French vineyards in spring between 2006 and 2012 in the “Biovigilance” project (Fried et al., 2008), covering three main wine production regions—Languedoc, Beaujolais, and northern Rhône valley and Champagne—along a latitudinal gradient of pedo‐climatic conditions and management practices (for a detailed description see (Fried et al., 2019)). Languedoc has a Mediterranean climate with a mean annual temperature of 14.1℃, and 686 mm annual rainfall in the surveyed plots (Hijmans et al., 2005) with a mean Treatment Frequency Index (TFI) for herbicides of 0.48, that is, the cumulative ratio of the dose applied to the recommended dose, for all herbicide treatments applied during the growing season. Beaujolais and northern Rhone valley have a semi‐continental climate with temperate influences, with a mean annual temperature of 11.4℃ and 776 mm annual rainfall with a mean TFI of 1.38. Finally, Champagne has a continental climate with oceanic influences, with a mean annual temperature of 10.1℃ and 657 mm annual rainfall with a mean TFI of 1.24.

Three main types of management practices can be distinguished: mowing (including crushing), soil tillage, and chemical treatments with herbicides. Different management practices or combinations are employed on the rank and the inter‐rank and management practices differ also on the same vineyard plot over the years. Thus, to summarize management practices of each year in each vineyard, we used the number of mowing, of soil tilling, and of herbicide treatments per year.

Forty‐six vineyards plots were surveyed: 18 plots in Languedoc, 18 plots in Beaujolais and northern Rhone valley, and 10 plots in Champagne. In each of the 46 vineyards plot, 2,000 m^2^ quadrat surveys were performed along rows and inter‐rows to account for different management practices. Following (Fried et al., 2019), we focused our analyses on the 30 most abundant species (Table S1). We extracted the presence and absence of standing weed species. Data from the presence or absence of species in the seed bank were not available and were therefore considered as a hidden variable and estimated with the probabilistic framework of HMMs (as described in the *Statistical analyses* session). We extracted seed mass values from the LEDA and TRY databases (Kattge et al., 2011, Kleyer et al., 2008). Seed mass, also called seed size, was defined as the oven‐dry mass of a species, expressed in mg. Mean seed mass was determined by weighing the total mass of between 20 and 100 individual seeds (depending on the species), then dividing the total dry weight by the number of seeds in the sample (Pérez‐Harguindeguy et al., 2013). Seed mass showed high variation among species with Asteraceae species and especially *Erigeron canadensis* having the lowest value (0.0001 g) and *Convolvulus arvensis* having the highest seed mass value (0.0145 g).

### 2.2 Statistical analyses

#### 2.2.1 Multilevel hidden Markov model (HMM)

Following Pluntz et al. (2018), we built a HMM by considering three states: “1” for both hidden state seeds and standing flora are absent in year *t*, “2” for seeds are present in year *t* but standing flora is absent in year *t* + 1, and “3” for seeds are present in year *t* and standing flora is present in year *t* + 1, underlying the two observations made when collecting data: (a) species not seen, (b) species seen. To specify the HMM proposed by Pluntz et al. (2018), we need some notations first.

- Observations (or events) are $X_{t}={\mathrm{flora}}_{t}$ and take values:
  0 is for species absent;
  1 is for species present.
- States are $Z_{t}=\left(S_{t‐1},X_{t}\right)$ with *S_t_* the state of the seed bank at *t*; the states *Z_t_* take values:
  1 is (0, 0) for both seeds and standing flora are absent;
  2 is (1, 0) for seeds are present but standing flora is absent on the following year;
  3 is (1, 1) for seeds are present, and then standing flora is present.
- The parameters we need are all probabilities:
  the germination probability *g*, which is the joint probability of seed germination success and of plant survival to adulthood,
  the probability of seed bank survival *s* from 1 year to the next without the addition of new seeds (seed survival constant through time),
  the probability of external colonization *c*, which is probability that at least one seed from outside arrived on the plot and survived to the onset of the next season) and
  the initialization parameter $p_{0}$, which is the probability that there were seeds in the soil the year before the first observation of the existing flora in the plot.

**FIGURE 1:**
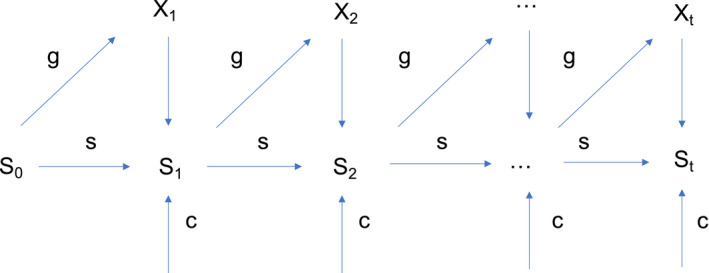
Graphical structure of the Hidden Markov Model (HMM) for weed species dynamics. Variable Xt represents the presence or absence of existing plants in the field at time *t*. Variable St is a hidden variable (never observed) that represents seeds in the seed bank at time *t*. The three parameters considered are the probability of seed survival in the soil (s), the probability of external colonization (c), and the probability of seed germination and survival until flowering (g). Figure from Pluntz et al. (2018) (license number from John Wiley and Sons 5080921466407)

The HMM then consists of three components (Figure 1), namely, the vector of initial states probabilities, the matrix of observation probabilities, and the matrix of transition probabilities:

- The vector of initial probabilities is (states in columns):

$$\left[\begin{array}{ccc} 1‐p_{0}\; & p_{0}\left(1‐g\right)\; & p_{0}g \end{array}\right];$$

- The matrix of observation probabilities is (states at t in rows, observations at t in columns):

$$\left[\begin{array}{cc} 1\; & 0\\ 1\; & 0\\ 0\; & 1 \end{array}\right];$$

- The matrix of transition probabilities is (states at t‐1 in rows, states at t in columns):

$$P=\left[\begin{array}{ccc} 1‐c & c\left(1‐g\right) & \mathrm{cg}\\ \left(1‐c\right)\left(1‐s\right) & \left(1‐g\right)\left(1‐\left(1‐c\right)\left(1‐s\right)\right) & g\left(1‐\left(1‐c\right)\left(1‐s\right)\right)\\ 0 & 1‐g & g \end{array}\right].$$

Last, we consider the effect of covariates on a demographic parameter say θ for g, s or c. We write for i the plot index and j the species index:

$$\begin{array}{c} \text{logit}\left(\theta \left(i,j\right)\right)=\alpha_{0}\left(j\right)+\alpha_{1}\left(j\right)\;\text{latitude}\left(i\right)+\alpha_{2}\left(j\right)\;\text{pH}\left(i\right)+\alpha_{3}\left(j\right)\;\text{silt}\left(i\right)+\alpha_{4}\left(j\right)\;\text{clay}\left(i\right)\\ +\alpha_{5}\left(j\right)\;\text{mowing}\left(i\right)+\alpha_{6}\left(j\right)\;\text{tillage}\left(i\right)+\alpha_{7}\left(j\right)\;\text{herbicide}\left(i\right) \end{array}$$

with the following random effects:

$$\alpha_{k}\left(j\right)∼N\left({\bar{\alpha }}_{k},\sigma_{k}^{2}\right),k=0,1,2,3,4$$

and

$$\alpha_{k}\left(j\right)∼N\left(\gamma_{k}+\beta_{k}\text{seedmass}\left(j\right),\sigma_{k}^{2}\right),k=5,6,7.$$

Note that there is a different model for each demographic parameter.

First, we tested at the plot level the effects on these demographic parameters of latitude and pedo‐climatic variables, namely, soil pH and soil texture with the proportion of silt and clay that were shown to be relevant in a previous study (Fried et al., 2019). Second, we assessed the effects of management practices on the demographic parameters at the species level using species random effects on both intercepts and slopes of these relationships, and an effect of seed mass on the slope of management practices. This hierarchical formulation of our model can also be interpreted as an interaction between a group indicator—seed mass—and individual‐level predictors—management practices (see chapter 13 in Gelman & Hill, 2007). Our multilevel HMM was fitted with a Bayesian approach using Markov Chain Monte Carlo (MCMC) simulations (code and data in Appendix S1). We used weakly informative normal prior distributions for the regression coefficients and uniform prior distributions for the standard deviation of the random effects. We ran two MCMC in parallel with different initial values, 10,000 iterations each, and an initial burn‐in of 2,500 iterations. We concluded to the significance of an effect if the 95% credible interval of the corresponding slope excluded zero. We also computed the proportion of explained variance for multilevel models (Gelman & Rubin, 1992).

## 3 RESULTS

### 3.1 Weed demography

Seed survival probability in the seed bank *s* varied from posterior mean 0.14 (*Anisantha sterilis*) to 0.82 (*Muscari neglectum*), germination probability *g* varied from posterior mean 0.13 (*Chenopodium album*) to 0.79 (*Diplotaxis erucoides*), and colonization probability *c* varied from posterior mean 0 (*Mercurialis annua*) to 0.86 (*Lamium amplexicaule*) (Appendix S1, Table S1).

### 3.2 Effects of pedo‐climatic factors on weed demography

**FIGURE 2:**
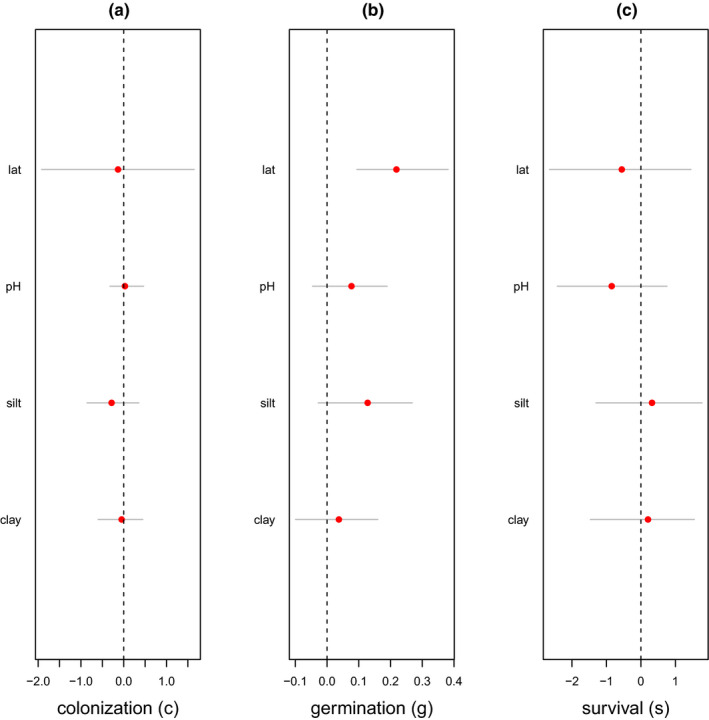
Effects of latitude and pedo‐climatic variables (soil pH, proportions of silt, and clay) on the demographic parameters of 30 weed species in French vineyards. Colonization (left panel), germination (middle panel), and survival (right panel) are considered. The red circles are the posterior means, and the thin grey lines are the 95% credible intervals. The effect was considered significant when the corresponding credible interval did not overlap 0 represented by the dashed vertical line

We found no significant effect of soil parameters and latitude on colonization *c* and seed survival probability *s* (Figure 2a, Figure 2c). Latitude had a significant effect on germination probability *g*, with species in plots from higher latitude having a higher germination probability (Figure 2b). Silt had a positive effect, albeit nonsignificant, on germination probability (Figure 2b).

### 3.3 Influence of seed mass on the effect of soil management practices on weed demography

**FIGURE 3:**
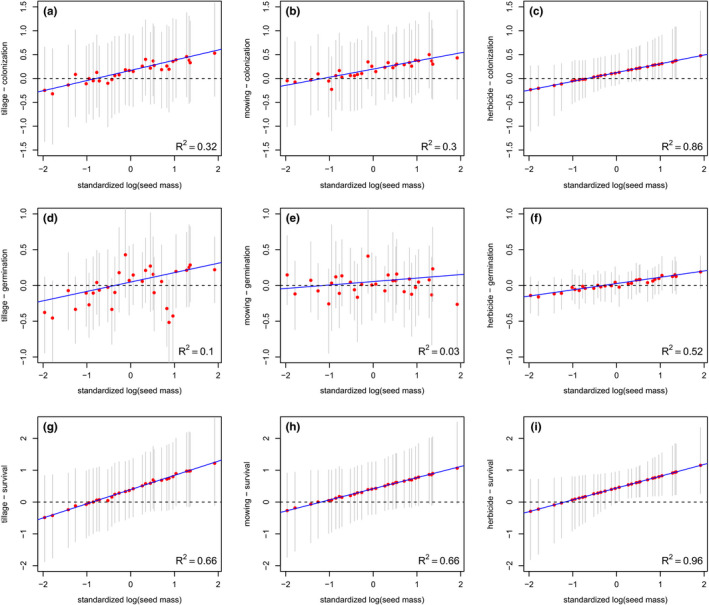
Slopes of the response of demographic parameter to management practices for 30 weed species in French vineyards. The effect of tillage (left column), mowing (middle column), and herbicides (right column) on colonization (upper row), germination (middle row), and survival (bottom row) is given as a function of seed mass on the log scale (blue solid line), as estimated by the multilevel hidden Markov model with species as a random effect, holding latitude, and pedo‐climatic variables to their mean values. In the mathematical terms, the blue solid line corresponds to $\gamma_{k}+\beta_{k}\mathrm{seedmass}$ with *k* an index for the management practice. The red circles are the posterior means, and the thin grey lines are the 95% credible intervals. The proportion of explained variance is also provided (bottom right corner in each panel)

Overall, seed mass explained interspecies variation in the effects of management practices on most of the demographic parameters (Figure 3), with a positive correlation between seed mass and the intensity of these effects (Table S2). Importantly, we also found that this correlation varied according to management practices.

The effect of the three management practices on colonization probability covaried positively with species seed mass (Figure 3a–c) with the strongest effect for herbicide application (*R* ^2^ = 0.86). Herbicide application enhanced more the rate of colonization for large‐seeded species, like *Malva sylvestris*, *Calendula arvensis*, *Anisantha sterilis,* or *Convolvulus arvensis* than small‐seeded species like *Erigeron canadensis* and *Cerastium glomeratum* (Figure 3c).

With regard to germination, the effect of herbicide application on vineyards covaried positively with seed mass: herbicide application increased more the germination probability of large‐seeded species than that of small‐seeded species (*R* ^2^ = 0.52; Figure 3f). The same result was found for the effect of tillage on germination probability (except for species *Erodium cicutarium, Fumaria officinalis, Muscari neglectum,* or *Cerastium glomeratum*) although the relationship was weak (*R* ^2^ = 0.1; Figure 3d). We found no significant relationship between seed mass and the effect of mowing on germination probability (Figure 3e).

Finally, the effects of the three management practices on survival probability were better explained by seed mass than germination and colonization. Seed survival in the seed bank increased more after tillage for large‐seeded species (except from *Erodium cicutarium, Fumaria officinalis, Muscari neglectum,* which also had a lower increase in germination probability after the tillage) (*R* ^2^ = 0.66; Figure 3h). We found the same pattern for the effect of mowing (*R* ^2^ = 0.66; Figure 3i) and herbicide (*R* ^2^ = 0.96; Figure 3j) with species like *Malva sylvestris* or *Calendula arvensis* showing a higher increase in survival in seed bank after mowing or herbicide application than *Erigeron canadensis, Cerastium glomeratum,* or *Cardamine hirsuta*.

## 4 DISCUSSION

### 4.1 Effect of management practices on demographic parameters

Disturbance is one of the most important environmental filters influencing vegetation structure and composition (Fried et al., 2019). In arable fields, the type of disturbances refers either to the physical disturbances that include soil tillage and mechanical weed control or to chemical disturbances such as herbicide treatments (Gaba et al., 2014). Frequent mechanical weed control is expected to reduce the abundance of species with short‐lived seeds and extend overall seed persistence (Albrecht & Auerswald, 2009), or accelerate the rate of seed mortality (Mohler, 1993). Mowing directly defoliates plants and can reduce growth, decrease plant survival and reduce or prevent seed production in two ways (Ferraro & Oesterheld, 2002). First, mowing changes the biotic environment, such as light, temperature, and soil moisture by disturbing mostly aboveground vegetation. Second, mowing changes competitive relationships between neighboring plants because different species die or regrow at different rates following mowing (Vilà & Terradas, 1998). Repeated chemical control in vineyards can be considered as an evolutionary force, due to weeds adaptation to different molecules. The positive effects of gaps in enhancing seedling recruitment are widely acknowledged (Chauhan et al., 2006; Turnbull et al., 1999).

Interestingly, our main finding was independent of the nature of farming practices and of the demographic process, such as colonization and survival in seed banks. When the number of disturbances increases (i.e., in plots with a higher number of herbicides, tillage, or mowing treatments), colonization and survival of large‐seeded species is selected, while these parameters are unchanged for small‐seeded species. In fact, having small seeds is a typical characteristic of species with a ruderal strategy (Grime, 1974), which are expected to be particularly adapted to disturbed environments and capable of colonizing freshly disturbed environments (Westoby et al., 2002). Our results show that for these species, the demographic parameters remain constant regardless of the level of disturbance. Their presence in the seed bank and in the emerged flora is therefore not modified by the intensity of the practices. This finding can be explained by the fact that larger seeds confer an advantage with higher seedling survival and germination probability under unfavorable environment (Marshall, 1986; Turnbull et al., 1999), at least on a relatively short term (Moles & Westoby, 2004) and a greater success of emerging from burial (Gardarin, Dürr, & Colbach, 2010; Gardarin et al., 2010) although they disperse less due to their larger seed mass (Fenner, 1998). We also found that tillage increased the probability of the success of colonization from an external source and the survival to the onset of the next season for large‐seeded species such as *Convolvulus arvensis*, *Lolium multiflorum*, *Cirsium arvense,* and *Anisantha sterilis*. One explanation is that inversion operations, like tillage, bury weed seeds at a depth where the seeds are less prone to predation and germination and thus persist longer (Mohler & Galford, 1997; Omami et al., 1999). Additionally, large‐seeded species are predicted to have reduced dormancy because their seedlings can draw on a larger food reserve, and hence establish in relatively unfavorable environments. Our results showed that herbicide application increased large‐seeded species germination probability and survival in seed bank. These results are in accordance with our first hypothesis that colonization should be more pronounced in frequently disturbed environments (Clark et al., 1999).

### 4.2 Implications for weed management in vineyards

Our results provide new perspectives in weed management by shedding light on the demography of the most “resistant” weeds to management practices. First, we demonstrated that frequent and intense disturbance such as tillage and herbicide management increases colonization of large‐seeded species. In terms of spatial management, these results will permit to identify which species can potentially spread from one field to the other or even from one inter‐row to the other. Second, most species with high colonization capacity are also characterized by medium dormancy. These species can persist in the landscape through both spatial movement and seed survival in the soil (Pluntz et al., 2018). Based on these findings, management practices can be advocated that prevent (a) weed species from producing new seeds (destruction before seeding) or (b) seed germination by planting mixtures of cover crops with germination synchronized with weed species and higher competitive abilities than weed species.

## 5 CONCLUSION

Alternative methods of weed control in vineyards are crucial not only for ensuring the sustainability and stability of farming systems, but also for providing important ecological services. However, in the last decades the vineyard has suffered an intensive management with a high mechanization (including frequent tilling) and/or use of herbicides, which affect species richness and abundance (Fried et al., 2019). Here, we used a novel statistical approach to determine the effect of management practices on seed bank dynamics in weed species. The strong relationships between these effects especially on seed survival and seed mass provide a reliable indicator for predicting the dynamics of weed communities. A promising avenue of research is to integrate into our modeling approach biotic filters such as the relative competitive ability of weeds and the interactions between weed species.

## CONFLICT OF INTEREST

None declared.

## AUTHOR CONTRIBUTIONS

**Elena Kazakou:** Conceptualization (lead); methodology (equal); project administration (lead); resources (equal); supervision (lead); writing–original draft (lead); writing–review and editing (lead). **Guillaume Fried:** Data curation (lead); resources (lead); writing–review and editing (equal). **Pierre‐Olivier Cheptou:** Methodology (equal); writing–review and editing (supporting). **Olivier Gimenez:** Conceptualization (equal); formal analysis (lead); funding acquisition (lead); methodology (lead); software (lead); validation (lead).

## Supporting information

Supplementary Material

## Data Availability

The data and code used in this study are available as Appendix S1.
