# Supplementary material for: Does seed mass drive interspecies variation in the effect of management practices on weed demography?
Source: Ecol Evol. 2021 Sep 2;11(19):13166–74. doi: 10.1002/ece3.8038 (PMC8495798; doi:10.1002/ece3.8038)
Supplement: Supplementary file 1 — Supplementary Material [file ECE3-11-13166-s001.docx]

**Electronic supplementary material**

## S1

To implement the Bayesian analysis of our multilevel HMM, we used non-informative normal prior distributions for the regression coefficients and uniform prior distributions for the standard deviation of the random effects. We ran two MCMC in parallel with different initial values, 10,000 iterations each and an initial burn-in of 2,500 iterations. We assessed convergence by visual inspection and by using the Gelman and Rubin R-hat diagnostic. We used OpenBUGS which allows parallel computation.

First, load the packages we need:

library(R2OpenBUGS) # bayesian analyses
library(coda) # bayesian diagnostics
library(mcmcplots) # viz mcmc
library(snow) # parallelization
library(snowfall) # parallelization
library(tidyverse) # data viz and manipulation

## ── Attaching packages ──────────────────────────────────────────────────────────────────────────────────────────────────────────────────────────────────── tidyverse 1.2.1 ──

## ✔ ggplot2 3.2.0 ✔ purrr 0.3.2.9000
## ✔ tibble 2.1.3 ✔ dplyr 0.8.3
## ✔ tidyr 1.0.0 ✔ stringr 1.4.0
## ✔ readr 1.3.1 ✔ forcats 0.4.0

## ── Conflicts ─────────────────────────────────────────────────────────────────────────────────────────────────────────────────────────────────────── tidyverse_conflicts() ──
## ✖ dplyr::filter() masks stats::filter()
## ✖ dplyr::lag() masks stats::lag()

We run two chains in parallel. To do so, we set the number of CPUs to two and assign the R2OpenBUGS library to each CPU:

sfInit(parallel=TRUE, cpus=2)

## Warning in searchCommandline(parallel, cpus = cpus, type = type,
## socketHosts = socketHosts, : Unknown option on commandline:
## rmarkdown::render('/Users/oliviergimenez/Desktop/appendix/
## appendix.Rmd',~+~~+~encoding~+~

## R Version: R version 3.6.1 (2019-07-05)

## snowfall 1.84-6.1 initialized (using snow 0.4-3): parallel execution on 2 CPUs.

sfLibrary(R2OpenBUGS)

## Library R2OpenBUGS loaded.

## Library R2OpenBUGS loaded in cluster.

Now get the data (available from <https://drive.google.com/file/d/1gzNfxnnGl8hL2czs15-T9KniwHV4bGg7/view?usp=sharing>) and format them for analysis in OpenBUGS:

load('dat.RData')
mydatax <- list(nsp = nsp, # number of species
 nsites = nsites, # number of plots
 nocc = nocc, # number of sampling occasions
 mydata = sp_histories_array, # detections/non-detections
 chim = chim, # herbicide
 mec = mec, # tillage
 fau = fau, # mowin
 lat = lat, # latitude
 seedmass = seedmass, # species seed mass
 ph = ph, # pH
 silt = silt, # % silt
 clay = clay) # % clay

Create separate directory for each CPU process:

folder1 <- paste(getwd(), "/chain1", sep="")
folder2 <- paste(getwd(), "/chain2", sep="")
##### dir.create(folder1); dir.create(folder2); # uncomment if you'd like to run the analysis
 # (warning: takes several hours)

Now specify the multilevel HMM model:

# sinking the model into a file in each directory
for (folder in c(folder1, folder2)){
 sink(paste(folder, "/nummodel.txt", sep=""))
cat("
 model{
 # DEFINE PARAMETERS

 # OBSERVATION PROCESS: probabilities of observations (columns) at a given occasion
 # given states (rows) at this occasion
 po[1,1] <- 1
 po[1,2] <- 0
 po[2,1] <- 1
 po[2,2] <- 0
 po[3,1] <- 0
 po[3,2] <- 1

 po.init[1,1] <- 1
 po.init[1,2] <- 0
 po.init[2,1] <- 1
 po.init[2,2] <- 0
 po.init[3,1] <- 0
 po.init[3,2] <- 1

 # STATE PROCESS: probabilities of states at t+1 (columns) given states at t (rows)

 # probabilities for each INITIAL STATES
 for (s in 1:nsp){ # for each species
 for (i in 1:nsites){ # for each site
 px0[s,i,1] <- 1 - p_knot[s]
 px0[s,i,2] <- p_knot[s] * (1 - gg[s,i])
 px0[s,i,3] <- p_knot[s] * gg[s,i]
 px[s,i,1,1] <- 1 - cc[s,i]
 px[s,i,1,2] <- (1 - gg[s,i]) * cc[s,i]
 px[s,i,1,3] <- gg[s,i] * cc[s,i]
 px[s,i,2,1] <- (1 - cc[s,i]) * (1 - ss[s,i])
 px[s,i,2,2] <- (1 - gg[s,i]) * (1 - (1 - cc[s,i]) * (1 - ss[s,i]))
 px[s,i,2,3] <- gg[s,i] * (1 - (1 - cc[s,i]) * (1 - ss[s,i]))
 px[s,i,3,1] <- 0
 px[s,i,3,2] <- 1 - gg[s,i]
 px[s,i,3,3] <- gg[s,i]
 } # end site
 } # end species

 # LIKELIHOOD

 for (s in 1:nsp){ # for each species
 for (i in 1:nsites){ # for each ind within species
 # estimated probabilities of initial states are the proportions
 # in each state at first capture occasion
 alive[s,i,1] ~ dcat(px0[s,i,1:3])
 mydata[s,i,1] ~ dcat(po.init[alive[s,i,1],1:2])
 for (j in 2:nocc){ # loop over time

 ## STATE EQUATIONS ##
 # draw states at j given states at j-1
 alive[s,i,j] ~ dcat(px[s,i,alive[s,i,j-1],1:3])

 ## OBSERVATION EQUATIONS ##
 # draw observations at j given states at j
 mydata[s,i,j] ~ dcat(po[alive[s,i,j],1:2])

 } # end time
 } # end ind
 } # end species

 # PRIORS
 for (s in 1:nsp){ # for each species
 logit(p_knot[s]) <- intpknot + epspknot[s]
 epspknot[s] ~ dnorm(0, tauknot)
 for (i in 1:nsites){ # for each ind within species
 logit(cc[s,i]) <- intercept[1,s] +
 slopelat[1,s] * lat[i] +
 slopeph[1,s] * ph[i] +
 slopesilt[1,s] * silt[i] +
 slopeclay[1,s] * clay[i] +
 slopemeca[1,s] * mec[i] +
 slopechim[1,s] * chim[i] +
 slopefau[1,s] * fau[i]
 logit(gg[s,i]) <- intercept[2,s] +
 slopelat[2,s] * lat[i] +
 slopeph[2,s] * ph[i] +
 slopesilt[2,s] * silt[i] +
 slopeclay[2,s] * clay[i] +
 slopemeca[2,s] * mec[i] +
 slopechim[2,s] * chim[i] +
 slopefau[2,s] * fau[i]
 logit(ss[s,i]) <- intercept[3,s] +
 slopelat[3,s] * lat[i] +
 slopeph[3,s] * ph[i] +
 slopesilt[3,s] * silt[i] +
 slopeclay[3,s] * clay[i] +
 slopemeca[3,s] * mec[i] +
 slopechim[3,s] * chim[i] +
 slopefau[3,s] * fau[i]
 }
 }

 intpknot ~ dnorm(0, 1)
 tauknot <- 1 / (sdpknot * sdpknot)
 sdpknot ~ dunif(0, 10)

 for (i in 1:3){ # for each parameter: cc, gg and ss

 meanint[i] ~ dnorm(0, 1)
 tauint[i] <- 1 / (sdint[i] * sdint[i])
 sdint[i] ~ dunif(0, 10)

 meanlat[i] ~ dnorm(0, 1)
 taulat[i] <- 1 / (sdlat[i] * sdlat[i])
 sdlat[i] ~ dunif(0, 10)

 meanph[i] ~ dnorm(0, 1)
 tauph[i] <- 1 / (sdph[i] * sdph[i])
 sdph[i] ~ dunif(0, 10)

 meansilt[i] ~ dnorm(0, 1)
 tausilt[i] <- 1 / (sdsilt[i] * sdsilt[i])
 sdsilt[i] ~ dunif(0, 10)

 meanclay[i] ~ dnorm(0, 1)
 tauclay[i] <- 1 / (sdclay[i] * sdclay[i])
 sdclay[i] ~ dunif(0, 10)

 ameca[i] ~ dunif(0, 1)
 bmeca[i] ~ dunif(0, 1)
 achim[i] ~ dunif(0, 1)
 bchim[i] ~ dunif(0, 1)
 afau[i] ~ dunif(0, 1)
 bfau[i] ~ dunif(0, 1)

 for (s in 1:nsp){ # for each species
 intercept[i,s] ~ dnorm(meanint[i], tauint[i])
 slopelat[i,s] ~ dnorm(meanlat[i], taulat[i])
 slopeph[i,s] ~ dnorm(meanph[i], tauph[i])
 slopesilt[i,s] ~ dnorm(meansilt[i], tausilt[i])
 slopeclay[i,s] ~ dnorm(meanclay[i], tauclay[i])

 slopemeca[i,s] ~ dnorm(slopemeca1[i,s], taumeca)
 slopemeca1[i,s] <- ameca[i] + bmeca[i] * seedmass[s]
 e.meca[i,s] <- slopemeca[i,s] - slopemeca1[i,s]

 slopechim[i,s] ~ dnorm(slopechim1[i,s], tauchim)
 slopechim1[i,s] <- achim[i] + bchim[i] * seedmass[s]
 e.chim[i,s] <- slopechim[i,s] - slopechim1[i,s]

 slopefau[i,s] ~ dnorm(slopefau1[i,s], taufau)
 slopefau1[i,s] <- afau[i] + bfau[i] * seedmass[s]
 e.fau[i,s] <- slopefau[i,s] - slopefau1[i,s]

 }
 }

taumeca <- 1 / (sdmeca * sdmeca)
sdmeca ~ dunif(0, 10)
tauchim <- 1 / (sdchim * sdchim)
sdchim ~ dunif(0, 10)
taufau <- 1 / (sdfau * sdfau)
sdfau ~ dunif(0, 10)

}
")
sink()
}

Define the function that will run MCMC on each CPU:

# Arguments:
# chain - will be 1 or 2
# x.data - the data list
# params - parameters to be monitored
parallel.bugs <- function(chain, x.data, params)
{
 # a. defining directory for each CPU
 sub.folder <- paste(getwd(),"/chain", chain, sep="")

 # b. specifying the initial MCMC values
 inits <- function()list(meanint = rnorm(3, 0, 1),
 meanlat = rnorm(3, 0, 1),
 meanph = rnorm(3, 0, 1),
 meansilt = rnorm(3, 0, 1),
 meanclay = rnorm(3, 0, 1))

 # c. calling OpenBugs
 bugs(data= x.data, inits=inits, parameters.to.save= params,
 n.iter = 10000, n.chains=1,
 model.file="nummodel.txt", debug=T, codaPkg=TRUE,
 useWINE=TRUE,
 OpenBUGS.pgm = "/Applications/OpenBUGS323/OpenBUGS.exe",
 working.directory = sub.folder,
 WINE="/usr/local/Cellar/wine/2.0.4/bin/wine",
 WINEPATH= "/usr/local/Cellar/wine/2.0.4/bin/winepath")
}

Then specify the parameters to be monitored:

parameters <- c("p_knot","intpknot","sdpknot","meanint","meanlat","meanph","meansilt",
 "meanclay","ameca","bmeca","achim","bchim","afau","bfau","intercept",
 "slopelat","slopeph","slopesilt","slopeclay","slopemeca","slopechim",
 "slopefau","sdint","sdlat","sdph","sdsilt","sdclay","sdmeca","sdchim",
 "sdfau","e.meca","e.chim","e.fau")

Now the code to fit the model. Note that we do not run it as the analysis takes several hours to complete.

# calling the sfLapply function that will run
# parallel.bugs on each of the 2 CPUs
start_time <- Sys.time()
sfLapply(1:2, fun=parallel.bugs, x.data=mydatax, params=parameters)
end_time <- Sys.time()
end_time - start_time #

For convenience, we provide the results and post-process them. You may download the MCMC outputs from <https://drive.google.com/file/d/1hYcYCLXkL4NEDS2S0FN8Qbkk3VmEg2z8/view?usp=sharing>, then load them in R:

load('MCMCoutputs.Rdata')

If you’d like to do it yourself, you’ll need the following lines of codes:

# locating position of each CODA chain and read them
chain1 <- paste(folder1, "/CODAchain1.txt", sep="")
chain2 <- paste(folder2, "/CODAchain1.txt", sep="")
res <- read.bugs(c(chain1, chain2), quiet=TRUE) # takes a minute or so
out2 <- as.mcmc(rbind(res[[1]],res[[2]]))
save(out2, file="MCMCoutputs.RData", compress="xz")

Rename relevant variables:

varnames(out2)[1:18] <- c("chim_cc","chim_gg","chim_ss","fau_cc","fau_gg","fau_ss","meca_cc",
 "meca_gg","meca_ss","slope_cc_chim","slope_gg_chim","slope_ss_chim",
 "slope_cc_fau","slope_gg_fau","slope_ss_fau","slope_cc_meca",
 "slope_gg_meca","slope_ss_meca")
varnames(out2)[381:383] <- c("clay_cc","clay_gg","clay_ss")
varnames(out2)[387:395] <- c("lat_cc","lat_gg","lat_ss","ph_cc","ph_gg","ph_ss","silt_cc",
 "silt_gg","silt_ss")

Get Figure 2:

posterior.medians <- apply(out2, 2, median)
#pdf('fig2.pdf')
par(mfrow=c(1,3))
caterplot(out2,
 parms = c("lat_cc","ph_cc","silt_cc","clay_cc"),
 collapse=FALSE,
 reorder = FALSE,
 style = 'plain',
 cex.labels = 1,
 labels = c( 'lat', 'pH', 'silt', 'clay'),
 quantiles = list(outer=c(0.025,0.975),
 inner=c(0.025,0.975)),
 col='gray',
 lwd=1)
caterpoints(posterior.medians[c("lat_cc","ph_cc","silt_cc","clay_cc")],
 pch=19,
 col="red")
abline(v = 0,
 col = 'black',
 lty = 2)
mtext('colonization (c)',
 side = 1,
 line = 3,
 cex = 1)
caterplot(out2,
 parms = c("lat_gg","ph_gg","silt_gg","clay_gg"),
 collapse=FALSE,
 reorder = FALSE,
 style = 'plain',
 cex.labels = 1,
 labels = c( 'lat', 'pH', 'silt', 'clay'),
 quantiles = list(outer=c(0.025,0.975),
 inner=c(0.025,0.975)),
 col='gray',
 lwd=1)
caterpoints(posterior.medians[c("lat_gg","ph_gg","silt_gg","clay_gg")],
 pch=19,
 col="red")
abline(v = 0,
 col = 'black',
 lty = 2)
mtext('germination (g)',
 side = 1,
 line = 3,
 cex = 1)
caterplot(out2,
 parms = c("lat_ss","ph_ss","silt_ss","clay_ss"),
 collapse=FALSE,
 reorder = FALSE,
 style = 'plain',
 cex.labels = 1,
 labels = c( 'lat', 'pH', 'silt', 'clay'),
 quantiles = list(outer=c(0.025,0.975),
 inner=c(0.025,0.975)),
 col='gray',
 lwd=1)
caterpoints(posterior.medians[c("lat_ss","ph_ss","silt_ss","clay_ss")],
 pch=19,
 col="red")
abline(v = 0,
 col = 'black',
 lty = 2)
mtext('survival (s)',
 side = 1,
 line = 3,
 cex = 1)


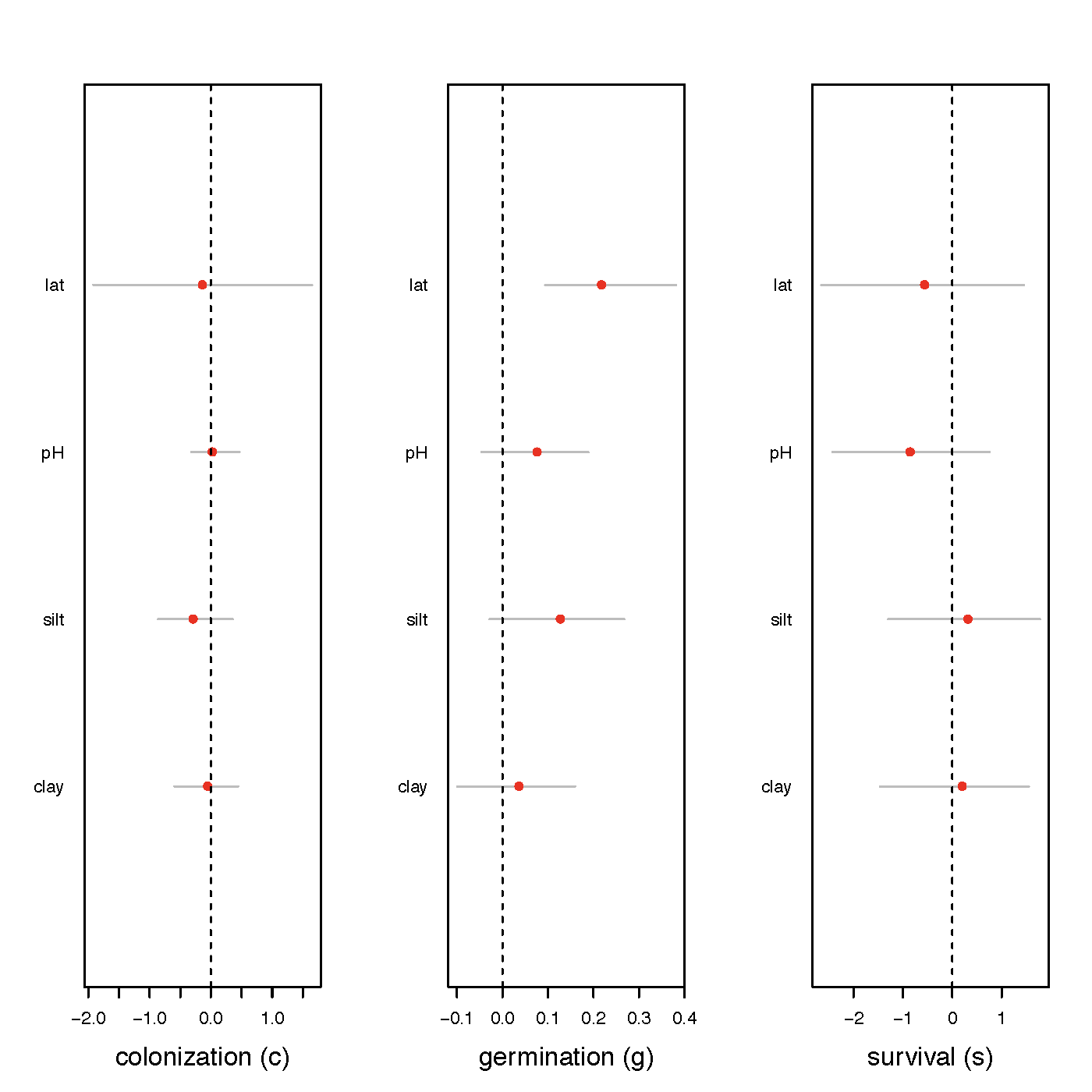


#dev.off()

Get proportion of explained variance:

names_col <- colnames(out2)

# R2 for meca
e.prac <- out2[,grep('e.meca',names_col)]
eprac <- vector("list", 3) # col, ger, sur
eprac[[1]] <- e.prac[,1:30]
eprac[[2]] <- e.prac[,31:60]
eprac[[3]] <- e.prac[,61:90]
slope <- out2[,grep('slopemeca',names_col)]
b <- vector("list", 3)
b[[1]] <- slope[,1:30]
b[[2]] <- slope[,31:60]
b[[3]] <- slope[,61:90]

rsquared.meca <- rep(NA,3)
#lambda.meca <- rep(NA,3)

for (i in 1:3){
 rsquared.meca[i] <- 1 - mean(apply (eprac[[i]], 1, var)) / mean (apply (b[[i]], 1, var))
}

# R2 for chim
e.prac <- out2[,grep('e.chim',names_col)]
eprac <- vector("list", 3) # col, ger, sur
eprac[[1]] <- e.prac[,1:30]
eprac[[2]] <- e.prac[,31:60]
eprac[[3]] <- e.prac[,61:90]
slope <- out2[,grep('slopechim',names_col)]
b <- vector("list", 3)
b[[1]] <- slope[,1:30]
b[[2]] <- slope[,31:60]
b[[3]] <- slope[,61:90]

rsquared.chim <- rep(NA,3)
#lambda.meca <- rep(NA,3)

for (i in 1:3){
 rsquared.chim[i] <- 1 - mean(apply (eprac[[i]], 1, var)) / mean (apply (b[[i]], 1, var))
}


# R2 for fau
e.prac <- out2[,grep('e.fau',names_col)]
eprac <- vector("list", 3) # col, ger, sur
eprac[[1]] <- e.prac[,1:30]
eprac[[2]] <- e.prac[,31:60]
eprac[[3]] <- e.prac[,61:90]
slope <- out2[,grep('slopefau',names_col)]
b <- vector("list", 3)
b[[1]] <- slope[,1:30]
b[[2]] <- slope[,31:60]
b[[3]] <- slope[,61:90]

rsquared.fau <- rep(NA,3)
#lambda.meca <- rep(NA,3)

for (i in 1:3){
 rsquared.fau[i] <- 1 - mean(apply (eprac[[i]], 1, var)) / mean (apply (b[[i]], 1, var))
}

rsquared.meca # R2 for tillage and 3 demographic parameters

## [1] 0.3197832 0.1028678 0.6617302

rsquared.chim # R2 for herbicide and 3 demographic parameters

## [1] 0.8584530 0.5220513 0.9600250

rsquared.fau # R2 for mowing and 3 demographic parameters

## [1] 0.29789503 -0.03217493 0.65964770

Last, get Figure 3:

species <- c('CONAR','CIRAR','SENVU','DIPER','GERRT','ERICA','TAROF','CVPSA','LACSE','SONOL',
 'VERPE','POAAN','EROCI','CHEAL','PLALA','STEME','MALSI','DAUCA','GERCO','FUMOF',
 'CARHI','PICEC','SONAS','MERAN','BROST','CERGL','LOLMU','MUSRA','LAMAM','CLDAR')
names <- species %>%
 enframe() %>%
 mutate(species = tolower(species)) %>%
 pull(species)
seedmassobs <- seedmass
ord <- order(seedmassobs)
names[ord]

## [1] "erica" "cergl" "carhi" "cvpsa" "diper" "senvu" "sonas" "sonol"
## [9] "poaan" "steme" "lacse" "lamam" "cheal" "tarof" "picec" "verpe"
## [17] "cirar" "plala" "meran" "gerrt" "dauca" "lolmu" "eroci" "fumof"
## [25] "musra" "gerco" "malsi" "cldar" "brost" "conar"

#pdf('fig3.pdf', width = 10, # The width of the plot in inches
# height = 10) # The height of the plot in inches

par(mfrow=c(3,3))
# cc and meca
fac <- out2[,grep("slopemeca\\[1,", varnames(out2))]
#fac <- rbind(fac[[1]],fac[[2]])
mean_species <- apply(fac,2,mean)
q25 <- apply(fac,2,quantile, probs = 2.5/100)
q975 <- apply(fac,2,quantile, probs = 97.5/100)
plot(seedmassobs[ord],
 mean_species[ord],
 type='n',
 ylim=c(-1.5,1.5),
xlab='standardized log(seed mass)',ylab='tillage - colonization',xaxt="n")
axis(1)
for (i in 1:30){
 segments(seedmassobs[ord][i],q25[ord][i],seedmassobs[ord][i],q975[ord][i],
 col='grey',pch=19,cex=0.5,lwd=0.6)
 points(seedmassobs[ord][i],mean_species[ord][i],col='red',cex=0.7, pch=19)
}
abline(c(mean(unlist(out2[,'meca_cc'])), mean(unlist(out2[,'slope_cc_meca']))), col = 'blue')
abline(h = 0, lty = 2)
text(1.5, -1.4, expression(R^2 == 0.32), cex = 1.2)

# cc and fau
fac <- out2[,grep("slopefau\\[1,", varnames(out2))]
#fac <- rbind(fac[[1]],fac[[2]])
mean_species <- apply(fac,2,mean)
q25 <- apply(fac,2,quantile, probs = 2.5/100)
q975 <- apply(fac,2,quantile, probs = 97.5/100)
plot(seedmassobs[ord],
 mean_species[ord],
 type='n',
 ylim=c(-1.5,1.5),
xlab='standardized log(seed mass)',ylab='mowing - colonization',xaxt="n")
axis(1)
for (i in 1:30){
 segments(seedmassobs[ord][i],q25[ord][i],seedmassobs[ord][i],q975[ord][i],
 col='grey',pch=19,cex=0.5,lwd=0.6)
 points(seedmassobs[ord][i],mean_species[ord][i],col='red',cex=0.7, pch=19)
}
abline(c(mean(unlist(out2[,'fau_cc'])), mean(unlist(out2[,'slope_cc_fau']))), col = 'blue')
abline(h = 0, lty = 2)
text(1.5, -1.4, expression(R^2 == 0.30), cex = 1.2)

# cc and chimie
fac <- out2[,grep("slopechim\\[1,", varnames(out2))]
#fac <- rbind(fac[[1]],fac[[2]])
mean_species <- apply(fac,2,mean)
q25 <- apply(fac,2,quantile, probs = 2.5/100)
q975 <- apply(fac,2,quantile, probs = 97.5/100)
plot(seedmassobs[ord],
 mean_species[ord],
 type='n',
 ylim=c(-1.5,1.5),
xlab='standardized log(seed mass)',ylab='herbicide - colonization',xaxt="n")
axis(1)
for (i in 1:30){
 segments(seedmassobs[ord][i],q25[ord][i],seedmassobs[ord][i],q975[ord][i],
 col='grey',pch=19,cex=0.5,lwd=0.6)
 points(seedmassobs[ord][i],mean_species[ord][i],col='red',cex=0.7, pch=19)
}
abline(c(mean(unlist(out2[,'chim_cc'])), mean(unlist(out2[,'slope_cc_chim']))), col = 'blue')
abline(h = 0, lty = 2)
text(1.5, -1.4, expression(R^2 == 0.86), cex = 1.2)

# gg and meca
fac <- out2[,grep("slopemeca\\[2,", varnames(out2))]
#fac <- rbind(fac[[1]],fac[[2]])
mean_species <- apply(fac,2,mean)
q25 <- apply(fac,2,quantile, probs = 2.5/100)
q975 <- apply(fac,2,quantile, probs = 97.5/100)
plot(seedmassobs[ord],
 mean_species[ord],
 type='n',
 ylim=c(-1,1),
xlab='standardized log(seed mass)',ylab='tillage - germination',xaxt="n")
axis(1)
for (i in 1:30){
 segments(seedmassobs[ord][i],q25[ord][i],seedmassobs[ord][i],q975[ord][i],
 col='grey',pch=19,cex=0.5,lwd=0.6)
 points(seedmassobs[ord][i],mean_species[ord][i],col='red',cex=0.7, pch=19)
}
abline(c(mean(unlist(out2[,'meca_gg'])), mean(unlist(out2[,'slope_gg_meca']))), col = 'blue')
abline(h = 0, lty = 2)
text(1.5, -0.9, expression(R^2 == 0.10), cex = 1.2)

# gg and fau
fac <- out2[,grep("slopefau\\[2,", varnames(out2))]
#fac <- rbind(fac[[1]],fac[[2]])
mean_species <- apply(fac,2,mean)
q25 <- apply(fac,2,quantile, probs = 2.5/100)
q975 <- apply(fac,2,quantile, probs = 97.5/100)
plot(seedmassobs[ord],
 mean_species[ord],
 type='n',
 ylim=c(-1,1),
xlab='standardized log(seed mass)',ylab='mowing - germination',xaxt="n")
axis(1)
for (i in 1:30){
 segments(seedmassobs[ord][i],q25[ord][i],seedmassobs[ord][i],q975[ord][i],
 col='grey',pch=19,cex=0.5,lwd=0.6)
 points(seedmassobs[ord][i],mean_species[ord][i],col='red',cex=0.7, pch=19)
}
abline(c(mean(unlist(out2[,'fau_gg'])), mean(unlist(out2[,'slope_gg_fau']))), col = 'blue')
abline(h = 0, lty = 2)
text(1.5, -0.9, expression(R^2 == 0.03), cex = 1.2)

# gg and chimie
fac <- out2[,grep("slopechim\\[2,", varnames(out2))]
#fac <- rbind(fac[[1]],fac[[2]])
mean_species <- apply(fac,2,mean)
q25 <- apply(fac,2,quantile, probs = 2.5/100)
q975 <- apply(fac,2,quantile, probs = 97.5/100)
plot(seedmassobs[ord],
 mean_species[ord],
 type='n',
 ylim=c(-1,1),
xlab='standardized log(seed mass)',ylab='herbicide - germination',xaxt="n")
axis(1)
for (i in 1:30){
 segments(seedmassobs[ord][i],q25[ord][i],seedmassobs[ord][i],q975[ord][i],
 col='grey',pch=19,cex=0.5,lwd=0.6)
 points(seedmassobs[ord][i],mean_species[ord][i],col='red',cex=0.7, pch=19)
}
abline(c(mean(unlist(out2[,'chim_gg'])), mean(unlist(out2[,'slope_gg_chim']))), col = 'blue')
abline(h = 0, lty = 2)
text(1.5, -0.9, expression(R^2 == 0.52), cex = 1.2)

# ss and meca
fac <- out2[,grep("slopemeca\\[3,", varnames(out2))]
#fac <- rbind(fac[[1]],fac[[2]])
mean_species <- apply(fac,2,mean)
q25 <- apply(fac,2,quantile, probs = 2.5/100)
q975 <- apply(fac,2,quantile, probs = 97.5/100)
plot(seedmassobs[ord],
 mean_species[ord],
 type='n',
 ylim=c(-2,2.5),
xlab='standardized log(seed mass)',ylab='tillage - survival',xaxt="n")
axis(1)
for (i in 1:30){
 segments(seedmassobs[ord][i],q25[ord][i],seedmassobs[ord][i],q975[ord][i],
 col='grey',pch=19,cex=0.5,lwd=0.6)
 points(seedmassobs[ord][i],mean_species[ord][i],col='red',cex=0.7, pch=19)
}
abline(c(mean(unlist(out2[,'meca_ss'])), mean(unlist(out2[,'slope_ss_meca']))), col = 'blue')
abline(h = 0, lty = 2)
text(1.5, -1.8, expression(R^2 == 0.66), cex = 1.2)

# ss and fau
fac <- out2[,grep("slopefau\\[3,", varnames(out2))]
#fac <- rbind(fac[[1]],fac[[2]])
mean_species <- apply(fac,2,mean)
q25 <- apply(fac,2,quantile, probs = 2.5/100)
q975 <- apply(fac,2,quantile, probs = 97.5/100)
plot(seedmassobs[ord],
 mean_species[ord],
 type='n',
 ylim=c(-2,2.5),
xlab='standardized log(seed mass)',ylab='mowing - survival',xaxt="n")
axis(1)
for (i in 1:30){
 segments(seedmassobs[ord][i],q25[ord][i],seedmassobs[ord][i],q975[ord][i],
 col='grey',pch=19,cex=0.5,lwd=0.6)
 points(seedmassobs[ord][i],mean_species[ord][i],col='red',cex=0.7, pch=19)
}
abline(c(mean(unlist(out2[,'fau_ss'])), mean(unlist(out2[,'slope_ss_fau']))), col = 'blue')
abline(h = 0, lty = 2)
text(1.5, -1.8, expression(R^2 == 0.66), cex = 1.2)

# ss and chimie
fac <- out2[,grep("slopechim\\[3,", varnames(out2))]
#fac <- rbind(fac[[1]],fac[[2]])
mean_species <- apply(fac,2,mean)
q25 <- apply(fac,2,quantile, probs = 2.5/100)
q975 <- apply(fac,2,quantile, probs = 97.5/100)
plot(seedmassobs[ord],
 mean_species[ord],
 type='n',
 ylim=c(-2,2.5),
xlab='standardized log(seed mass)',ylab='herbicide - survival',xaxt="n")
axis(1)
for (i in 1:30){
 segments(seedmassobs[ord][i],q25[ord][i],seedmassobs[ord][i],q975[ord][i],
 col='grey',pch=19,cex=0.5,lwd=0.6)
 points(seedmassobs[ord][i],mean_species[ord][i],col='red',cex=0.7, pch=19)
}
abline(c(mean(unlist(out2[,'chim_ss'])), mean(unlist(out2[,'slope_ss_chim']))), col = 'blue')
abline(h = 0, lty = 2)
text(1.5, -1.8, expression(R^2 == 0.96), cex = 1.2)


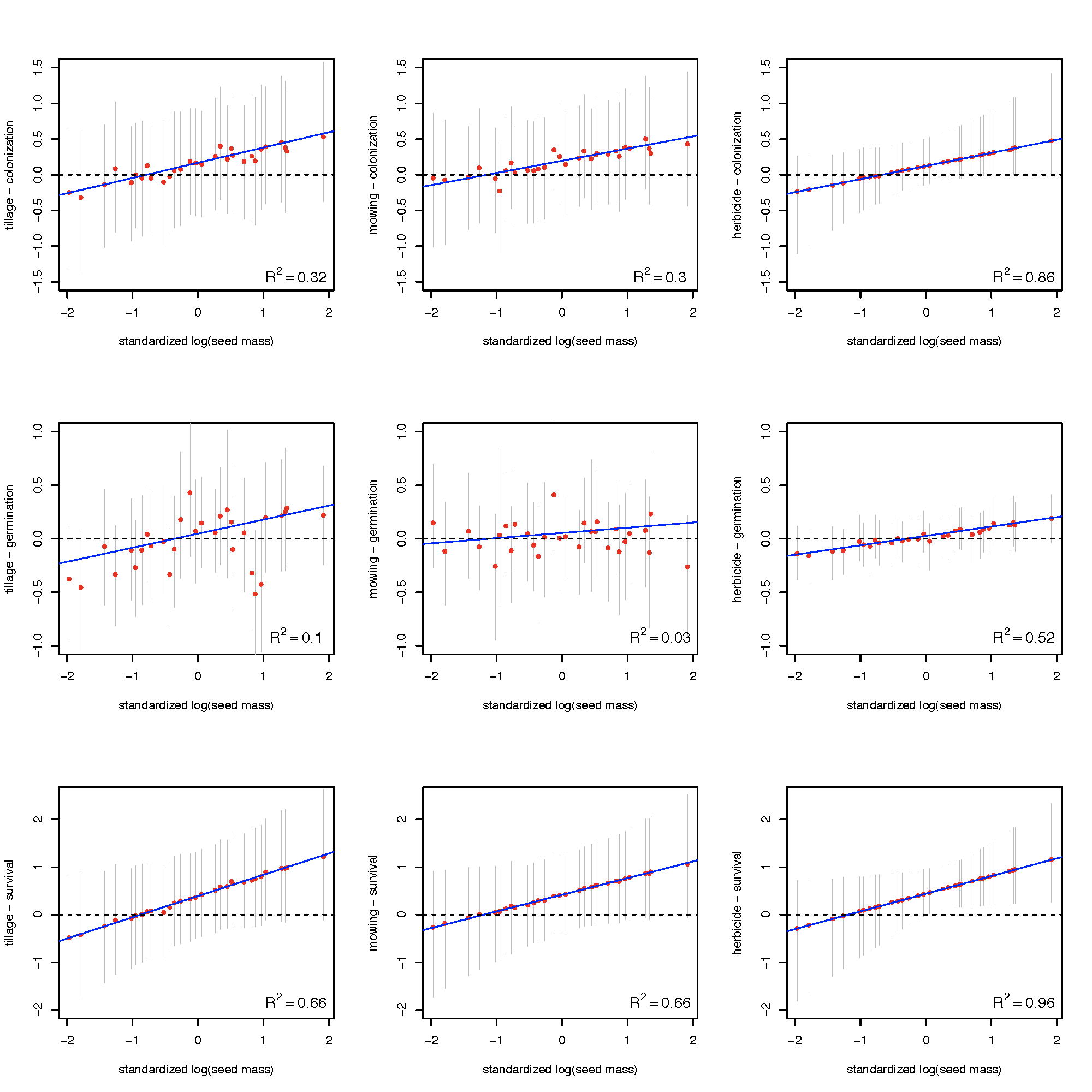


#dev.off()

Get posterior means and credible intervals for the $\beta$’s.

mean(unlist(out2[,'slope_cc_meca']))

## [1] 0.213291

mean(unlist(out2[,'slope_cc_fau']))

## [1] 0.1702896

mean(unlist(out2[,'slope_cc_chim']))

## [1] 0.1839739

mean(unlist(out2[,'slope_gg_meca']))

## [1] 0.1312281

mean(unlist(out2[,'slope_gg_fau']))

## [1] 0.04848915

mean(unlist(out2[,'slope_gg_chim']))

## [1] 0.08775498

mean(unlist(out2[,'slope_ss_meca']))

## [1] 0.4462417

mean(unlist(out2[,'slope_ss_fau']))

## [1] 0.3499389

mean(unlist(out2[,'slope_ss_chim']))

## [1] 0.3713638

round(quantile(unlist(out2[,'slope_cc_meca']), probs = c(2.5,97.5)/100),2)

## 2.5% 97.5%
## 0.01 0.64

round(quantile(unlist(out2[,'slope_cc_fau']), probs = c(2.5,97.5)/100),2)

## 2.5% 97.5%
## 0.01 0.54

round(quantile(unlist(out2[,'slope_cc_chim']), probs = c(2.5,97.5)/100),2)

## 2.5% 97.5%
## 0.00 0.61

round(quantile(unlist(out2[,'slope_gg_meca']), probs = c(2.5,97.5)/100),2)

## 2.5% 97.5%
## 0.01 0.31

round(quantile(unlist(out2[,'slope_gg_fau']), probs = c(2.5,97.5)/100),2)

## 2.5% 97.5%
## 0.00 0.16

round(quantile(unlist(out2[,'slope_gg_chim']), probs = c(2.5,97.5)/100),2)

## 2.5% 97.5%
## 0.01 0.20

round(quantile(unlist(out2[,'slope_ss_meca']), probs = c(2.5,97.5)/100),2)

## 2.5% 97.5%
## 0.02 0.96

round(quantile(unlist(out2[,'slope_ss_fau']), probs = c(2.5,97.5)/100),2)

## 2.5% 97.5%
## 0.01 0.95

round(quantile(unlist(out2[,'slope_ss_chim']), probs = c(2.5,97.5)/100),2)

## 2.5% 97.5%
## 0.02 0.98

**Table S1:** Parameter estimates from the Biovigilance dataset. Posterior means are provided.

| Species name | germination | colonization | survival |
| --- | --- | --- | --- |
| *Alopecurus myosuroides* | 0.59 | 0.09 | 0.51 |
| *Amaranthus retroflexus* | 0.35 | 0.00 | 0.91 |
| *Anagallis arvensis* | 0.17 | 0.00 | 0.92 |
| *Aphanes arvensis* | 0.34 | 0.01 | 1.00 |
| *Atriplex patula* | 0.21 | 0.00 | 0.88 |
| *Brassica napus* | 0.27 | 0.09 | 0.81 |
| *Calystegia sepium* | 0.55 | 0.09 | 0.71 |
| *Capsella bursa-pastoris* | 0.34 | 0.04 | 1.00 |
| *Chenopodium album* | 0.57 | 0.16 | 0.81 |
| *Cirsium arvense* | 0.37 | 0.25 | 0.75 |
| *Convolvulus arvensis* | 0.40 | 0.17 | 0.86 |
| *Digitaria sanguinalis* | 0.41 | 0.05 | 0.77 |
| *Echinochloa crus-galli* | 0.53 | 0.11 | 0.84 |
| *Euphorbia helioscopia* | 0.30 | 0.06 | 1.00 |
| *Fumaria officinalis* | 0.29 | 0.08 | 0.83 |
| *Galium aparine* | 0.56 | 0.20 | 0.76 |
| *Geranium dissectum* | 0.32 | 0.01 | 0.98 |
| *Lactuca serriola* | 0.57 | 0.06 | 0.35 |
| *Lamium purpureum* | 0.35 | 0.03 | 1.00 |
| *Lolium perenne* | 0.45 | 0.11 | 0.70 |
| *Matricaria chamomilla* | 0.51 | 0.13 | 0.18 |
| *Mercurialis annua* | 0.48 | 0.03 | 1.00 |
| *Papaver rhoeas* | 0.41 | 0.03 | 0.95 |
| *Poa annua* | 0.47 | 0.07 | 0.77 |
| *Polygonum aviculare* | 0.40 | 0.05 | 0.91 |
| *Polygonum convolvulus* | 0.37 | 0.13 | 0.92 |
| *Polygonum persicaria* | 0.33 | 0.00 | 0.85 |
| *Raphanus raphanistrum* | 0.33 | 0.11 | 0.64 |
| *Senecio vulgaris* | 0.52 | 0.36 | 0.63 |
| *Sinapis arvensis* | 0.56 | 0.12 | 0.65 |
| *Solanum nigrum* | 0.35 | 0.14 | 0.89 |
| *Sonchus asper* | 0.30 | 0.08 | 0.97 |
| *Sonchus oleraceus* | 0.29 | 0.24 | 0.50 |
| *Stellaria media* | 0.48 | 0.07 | 0.85 |
| *Taraxacum officinale* | 0.30 | 0.22 | 0.20 |
| *Veronica hederaefolia* | 0.45 | 0.04 | 1.00 |
| *Veronica persicaria* | 0.43 | 0.12 | 0.96 |
| *Viola arvensis* | 0.54 | 0.04 | 0.80 |

**Table S2:** Parameter estimates of the slope of the relationship between seed mass and the intensity of management practices on demographic parameters. These parameters are denoted $\beta_{k}$in the main text, and correspond to the slope of the blue line in Figure 3. Posterior means and 95% credible intervals are reported.

|  | **Tillage** | **Mowing** | **Herbicide** |
| --- | --- | --- | --- |
| **Colonization** | 0.21 (0.01, 0.64) | 0.17 (0.01, 0.54) | 0.18 (0.00, 0.61) |
| **Germination** | 0.13 (0.01, 0.31) | 0.05 (0.00, 0.16) | 0.09 (0.01, 0.20) |
| **Survival** | 0.45 (0.02, 0.96) | 0.35 (0.01, 0.95) | 0.35 (0.02, 0.98) |
